# Kinetic and Mechanistic Analysis of Phenol Adsorption on Activated Carbons from Kenaf

**DOI:** 10.3390/molecules29204941

**Published:** 2024-10-18

**Authors:** Delia Omenat-Morán, Carlos J. Durán-Valle, Manuel A. Martínez-Cañas

**Affiliations:** 1 Instituto del Corcho, la Madera y el Carbón Vegetal (CICYTEX), Pol. Ind. El Prado, c/Pamplona s/n, 06800 Mérida, Spain; delia.omenat@juntaex.es; 2 IACYS, Universidad de Extremadura, Avda. de Elvas, s/n, 06006 Badajoz, Spain; 3 Centre for Scientific Research and Technology in Extremadura, Technological AgriFood Institute, Government of Extremadura, Av. Adolfo Suárez s/n, 06007 Badajoz, Spain; manuel.martinez@juntaex.es

**Keywords:** activated carbon, kenaf, adsorption, DFT, phenol, kinetic

## Abstract

Activated carbons were prepared from kenaf (*Hibiscus cannabinus* L.). Carbonization was carried out at 600 °C for 2 h, and activation was performed using air at 600 °C and using CO_2_ at 750 °C. The activated carbons obtained were treated with HNO_3_ and H_2_SO_4_. The samples were characterized by their chemical and physical structure. The activated carbons obtained were mainly macroporous, and their structure underwent major changes with the activation method and acid treatment. Activated carbons were alkaline and acid-treated carbons were neutral. They were used for phenol adsorption and a kinetic and mechanistic study of adsorption was carried out. The fit to the pseudo-second order and Elovich models was predominant. The rate-limiting step of the process was determined to be diffusion within the pores, as the experimental data fit the Bangham model. DFT simulation showed that the preferred adsorption position involves π-π stacking and that oxidation enhances this interaction. Furthermore, the simulation showed that the interaction of phenol with oxygenated functional groups depends on the type of functional group.

## 1. Introduction

Many biomass types may be used as raw materials to produce activated carbons. Numerous recent reviews on this topic provide lists of biomass materials used for this purpose. Some of these reviews are specific to a particular material type, raw material, preparation method or application, i.e., the preparation of activated carbon spheres [1] or composites [2], the use of biowastes [3,4], food waste [5] or spent tires [6], pyrolytic [7] or hydrothermal carbonization [8,9], application to carbon electrodes [10], adsorption of formaldehyde [11], dyes [12], or perfluorinated compounds [13].

Biomass is a renewable resource, accessible in many parts of the world and of varied origins. To obtain activated carbon, it is essential to select both the precursor and the carbonization and activation methods [7]. It should be considered that both textural properties and surface chemistry can influence the activity of these materials [14], so their study remains a topic of great interest.

The kenaf plant (*Hibiscus cannabinus* L.) has attracted attention as a source of natural fiber, with both ecological and economic advantages, because it grows rapidly [15,16]. It is a plant native to Africa that grows in high densities and can be adapted to other parts of the world. Under good conditions, kenaf grows up to a height of nearly 4 m in 5–6 months [17].

The outer stem bark contains soft and long fibers (bast), and the center of the stem is formed by short fibers (core). Although both contain fibers, it has been shown that the inside of kenaf has different properties for the outer skin.

The traditional use of kenaf is focused on the production of its outer fiber, e.g., for making ropes, sacs, canvases, and carpets [18]. However, new applications of kenaf have recently been developed, such as adsorbent material for oils and liquids [16], thermal and acoustic insulation in composite boards [15,19], automotive components [20], cementitious material components [21], and even as a source of natural chemicals [16,22]. These uses of external fiber result in a considerable amount of internal fiber with no obvious destination.

Although kenaf core fiber has some applications as animal bedding [16], it makes up most of the stem. Most of the production is discarded and may be considered as waste [23]. Its transformation into a useful product such as activated carbon would avoid the problem of its disposal, in addition to being a zero-cost material. Since kenaf core fiber is characterized by relatively high lignin and low ashes content [17], it is a promising raw material for producing high-quality activated carbon commercially to derive better economic value.

In the present study, activated carbon was prepared from kenaf core fiber. The activation process was carried out in two different manners: one with air and one with CO_2_. Some authors have also prepared activated carbon from this material. It has been used as a gas [24,25], organic [26,27], or heavy metals [28,29] adsorbent.

In addition, carbon oxidation treatments using sulfuric acid and nitric acid were studied.

The activation processes and the oxidative treatments were evaluated by characterizing their physical and chemical structure.

The kinetics of phenol adsorption were studied using different concentrations of carbon applying different kinetic models. These experiments and theoretical studies using DFT methods have allowed us to study the mechanism of phenol adsorption. Phenol is the simplest molecule of the phenol family. It is a significant contributor to water pollution [30] and is produced in natural or human activities [31]. For this reason, it is frequently found in drinking water sources. Moreover, phenols are characterized by the fact that they are often recalcitrant compounds, difficult to oxidize by classical methods, and, in many cases, poorly biodegradable. This compound was selected because the simplicity of its structure with only one functional group allows a simplified study especially in terms of theoretical simulation.

Also, phenol is a representative organic molecule for many possible pollutants or chemicals of interest that present aromatic structures accompanied by some polar functional groups, such as dyes, drugs, etc. Although there are some precedents for phenol adsorption with kenaf-activated carbons, these have been prepared by mixing the two types of fiber and using other preparation conditions [32,33,34].

This research work investigates the use of a waste material (kenaf internal fiber) to prepare activated carbon as an adsorbent, which we are unaware of having been used individually before. The activated carbon has also been modified with mineral acids and the materials obtained have been extensively characterized. To this experimental work, we have added an extensive theoretical study on the adsorption mechanism by means of kinetic and DFT models that provide information on the energy of the adsorption process and the influence of oxygenated functional groups on phenol adsorption.

## 2. Results and Discussion

Table 1 shows an abstract of the adsorbents used and how they are prepared.

### 2.1. Physical Structure

#### 2.1.1. Surface Area and Pore Size Distribution

**Figure 1:**
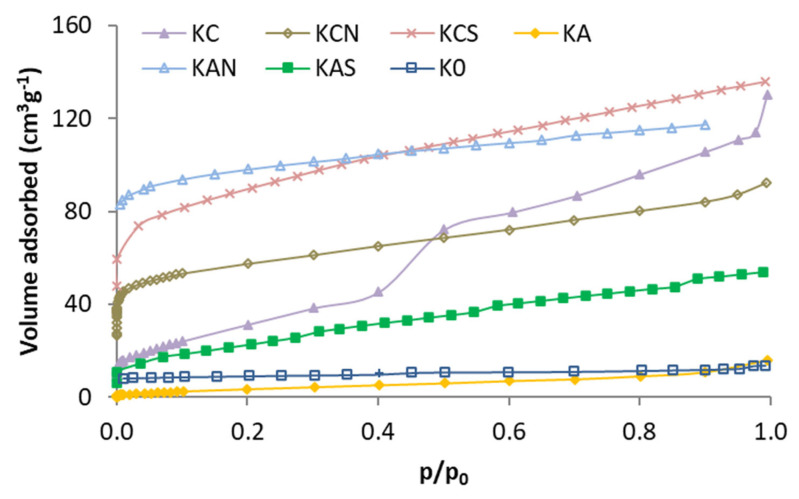
Nitrogen adsorption isotherms of the kenaf carbons.

The N_2_ adsorption isotherms of the activated kenaf carbons are shown in Figure 1. As shown in isotherms, carbonized kenaf has a poorly developed porosity, whereas all the activated carbons show a greater porosity (except KA). Isotherms with higher capacity are of type I (KAN, KCS, and KCN), type II (KAS), or type I + II (KC) [35]. The KC isotherm has a “step” in the middle of the isotherm that can be a significant volume of pores with a narrow distribution. This is not observed in the KC acid-treated carbons (KCN and KCS). Treatment with mineral acids produced a different effect on the two activated carbons. While treatment with H_2_SO_4_ showed the best result produced on carbon activated with CO_2_ (KCS), treatment with HNO_3_ is best suited for the one activated with air (KAN).

**Figure 2:**
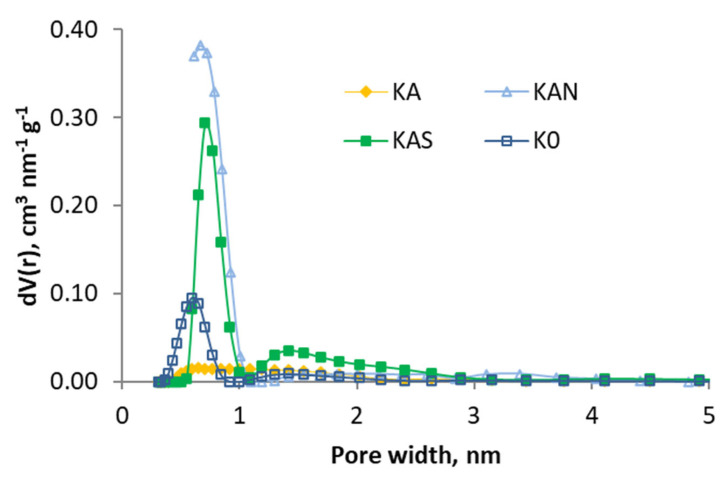
Pore size distributions (based in DFT) of the samples activated with air and K0.

**Figure 3:**
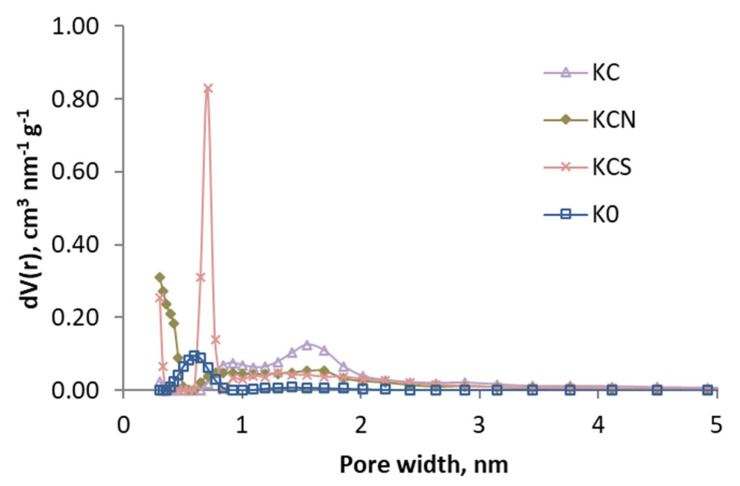
Pore size distributions (based in DFT) of the samples activated with CO_2_ and K0.

In Figure 2 and Figure 3, the pore size distribution is shown by applying the DFT model, and it can be observed that the effect of the treatments is different: the activation caused a loss of the narrow porosity, which was regained (at a different width) with acid oxidation treatments.

By applying the BET, Dubinin–Radushkevich, and t-plot methods to the adsorption data, the values of the specific surface area (S_BET_), micropore volume (W_0_), adsorption energy (E_0_), and average half-pore width (L) were calculated. Such values are listed in Table 2.

In the kenaf carbons, the S_BET_ and the micropore volume increased considerably with the activation and oxidation treatments, except in the KA sample. The S_BET_ data, so low and similar to the initial data shown by the carbonized KA sample, may be due to a blockage of the porosity in the sample caused by: (i) products generated during the carbonization process and which have not been eliminated by activation with air; or (ii) by ash produced in the activation with air. Both can be eliminated in the acid treatment.

The activation treatment with CO_2_ (KC sample) had a limited effect on micropores: the volume increased nearly twice, but the adsorption energy (E_0_) and average diameter (L) were similar. However, it differed greatly with air activation (KA sample): micropores almost disappeared in KA, and the average diameter widened. This may be because the blocked pores are the narrowest, so the adsorption energy is lower as they do not have micropores, and the average width increases as the blocked pores cannot be measured.

The volume of meso- and macropores was determined by mercury porosimetry and is shown in Table 2. All kenaf samples showed a width distribution of porosity (except micropores in KA) and were essentially macroporous.

The mesopore volume was higher in the carbons of the KA series than in the KC series and was higher when sulfuric acid was used compared to nitric acid. The macropore volume, in contrast, was higher in the KC series than in the KA series and was also greater when sulfuric acid was used.

In the carbons from kenaf activated with air, the less developed wide porosity (meso and macropores) corresponds to activated carbon KAN, which was the furthest developed narrow porosity. In the activated carbons with CO_2_, the acid treatment decreased the macropore volume and increased the mesopore volume.

#### 2.1.2. Scanning Electron Micrograph

Figure S1 shows SEM images of some samples. Despite undergoing the process of carbonization, activation, and oxidation, the carbons preserved the initial cellular structure corresponding to plant biochar.

### 2.2. Chemical Structure

#### 2.2.1. Elemental Analysis

When the activation process occurs in kenaf biochar, a slight decrease in oxygen content can be observed since heat treatment eliminates thermally labile functional groups, such as some that contain oxygen. Although in the case of KA the activation is carried out with oxygen, the possible formation of oxygenated groups is less than the destruction of these groups by the effect of temperature.

On the contrary, the oxidation treatment produces an increased oxygen content in the samples, although the amount gained depends on the method and precursor used [36,37]. This effect is observed in the results shown in Table 3 where the oxidation treatments produced, in general, an increase in the oxygen content of the activated carbons, except for KCS. Besides increasing the oxygen content, this is greater when HNO_3_ is used than when H_2_SO_4_ is used, which has also been reported by other authors who compared HNO_3_ with other oxidizing agents [38,39]. The increased oxygen content can be attributed to the formation of functional groups with oxygen-carbon bonds, as well as to the formation of -NO_2_ groups, although it is difficult to assert that this would happen in all cases [39].

HNO_3_ treatment also slightly increases nitrogen content, possibly due to remnants of the acid or any of its by-products or carbon nitration. Some authors [40,41] have attributed this observation to the formation of nitro and nitrate aromatic compounds.

Likewise, treatment with H_2_SO_4_ produces a slight increase in the sulfur content of the carbon samples treated, as seen in Table 3. This may be due to the same reasons as indicated for nitrogen, although a better explanation is the existence of sulfonic acid groups covalently bonded to carbon [42,43].

#### 2.2.2. Proximate Analysis

According to the results shown in Table 3, a slight increase in the ash content with activation treatments was observed due to a loss of organic material in the process.

The oxidation treatment increases the volatile matter in all samples except KCS. This effect is more pronounced when nitric acid is used.

The increase in volatile matter can be attributed to the functional groups formed with the oxidation treatment. These functional groups are thermally labile and, therefore, constitute an important part of the volatile matter. This result is consistent with the variation in oxygen content. Concentrated acids may also degrade functional groups if they are not stable. This fact may explain the decrease in volatile matter in the KCS sample because the less oxidizing, more strongly acidic, and higher concentration compound is used. The degradation effect is, in this case, greater than the oxidation effect. Since this is not the case for KAS, it must be assumed that functional groups of different types must be formed during activation.

Another effect that is usually noticed is that the acid treatment leaches the mineral matter from the activated carbons.

#### 2.2.3. X-Ray Photoelectron Spectroscopy

Table 4 shows the results of the elemental analysis determined by XPS analysis.

Acid treatments only increase oxygen content in those treated with nitric acid.

Furthermore, the nitrogen content increased in the samples treated with nitric acid (KAN and KCN) and the sulfur content increased in the samples treated with sulfuric acid (KAS and KCS).

In general, both types of elemental analysis are consistent in their results, indicating that the effect produced by different processes on the material’s surface (Table 4) was similar to the effect produced in the entire material as a bulk (Table 3).

The components of these spectra are assigned based on databases [44,45,46] and literature references [47].

As shown in Table 5, the activation process in carbons induced a slight increase in oxidized forms. The acid treatment produces a significant increase at the peak near 290 eV and a smaller decrease at the peak near 286 eV. No significant component has been detected at medium values of B.E. This can be explained by the fact that carbonyl groups (the main functional group of this component) are usually labile and must be lost in the carbonization step. Subsequent oxidation, when carried out in acid or aqueous acidic media, may form hydrogen-containing oxygenated groups [36] but is not likely to create new carbonyl groups, which are also alkaline and could react with acids.

Two zones can be differentiated in the XPS spectrum of N 1s (Table 6). The first zone is around 398–401 eV and can be regarded as characteristic of pyrrolic nitrogen, pyridones, quaternary nitrogen, and other reduced forms of nitrogen. The second zone usually appears around 405 eV and is characteristic of oxidized nitrogen functionalities.

There are also two different zones in the case of sulfur (Table 6). One appears around 163–165 eV and is usually attributed to SH groups attached to the phenol ring and sulfur bonded to the carbon in the structures C-S-C and R-S-S-OR. The other zone ranges from 168 to 169 eV and is attributed to forms of sulfur in a high oxidation state, such as sulfates, sulfites, and sulfonic acids.

**Figure 4:**
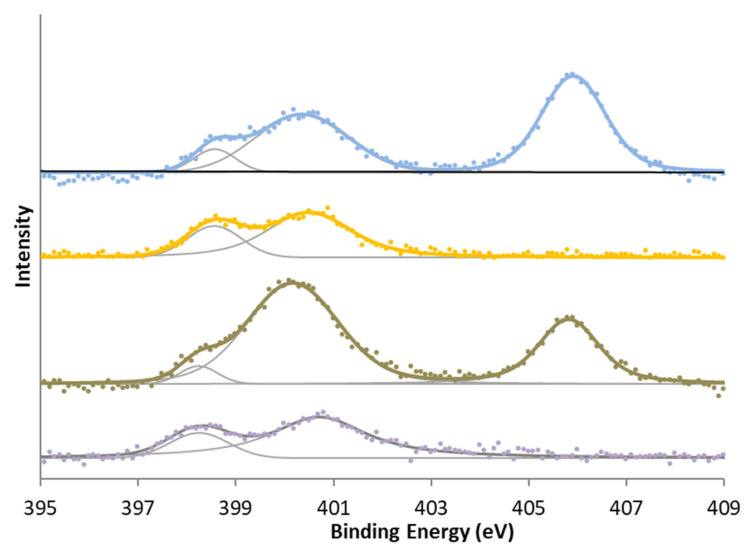
N 1s XPS spectra for (bottom to top) KC, KCN, KA, and KAN.

**Figure 5:**
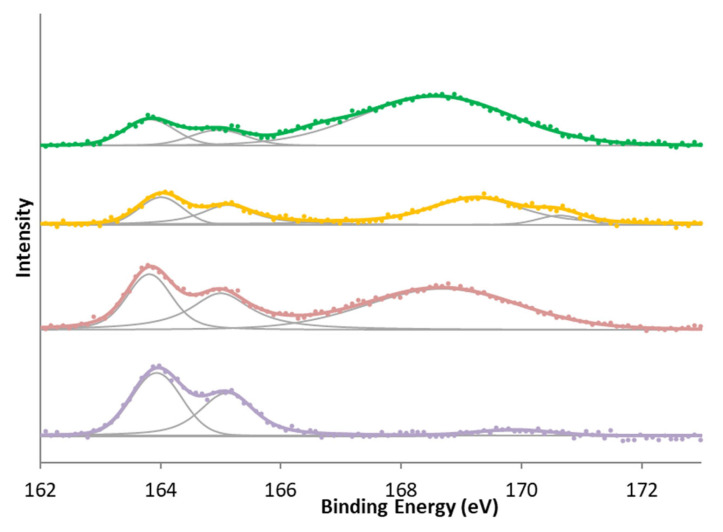
S 2p XPS spectra for (bottom to top) KC, KCS, KA, and KAS.

In surface nitrogen and sulfur groups, the most significant differences were observed when oxidation treatment was given to the samples. In the case of nitrogen, all samples show the reduced form of nitrogen, whereas in the case of modification with nitric acid carbons, a second characteristic peak group from oxidized nitrogen or -NO_2_ groups is also shown in Figure 4. In the case of sulfur (Figure 5), there is initially a high amount of sulfur in a high oxidation state. This is reduced in the activation treatment, but treatment with sulfuric acid increases the proportion of oxidized sulfur again. This has mainly been attributed [39,40,41,48,49] to the formation of sulphonic groups, although other sulfur functional groups may have formed.

#### 2.2.4. Point of Zero Charge (PZC) and Total Surface Acidity and Basicity

Table 7 shows the PZC results of different samples and acid/basic groups determined by titration. Oxidized carbons have a lower PZC than other samples. In kenaf carbons, there are no differences according to the acid used, while in commercial activated carbons, there are significant differences between nitric and sulfuric acid [39].

The adsorbents whose PZC is basic have a higher contribution of basic groups on their surface, whereas those who have a PZC near neutral pH show a similar contribution of acidic and basic groups. In addition, the activation process significantly increased the number of alkaline groups on the carbon surface. The total acid-active sites on the surface increased with the oxidation treatment, whereas the total basic active sites decreased.

The most significant number of acid-active sites was obtained after treatment with nitric acid, as this fixed the largest amount of oxygen. Interestingly, the activated carbon treated with sulfuric acid was as acidic as the one treated with nitric acid but with less acidic functional groups. This can be explained by the formation of sulfonic acid groups, which are more acidic than carboxyl or phenol/hydroxyl groups.

### 2.3. Kinetic Experiments

#### 2.3.1. Kinetic Models

**Figure 6:**
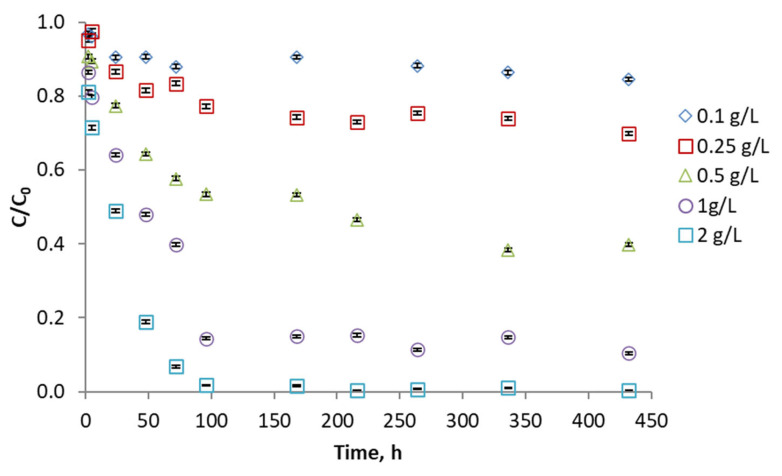
Phenol adsorption kinetics with KCN activated carbon using different proportions of adsorbent.

The evaluation of phenol concentration was studied using different adsorbent ratios. As expected, the higher the amount of activated carbon used, the better the pollutant removal. More than 90% of the phenol is removed with all adsorbents except the less porous ones (KA and KAS) where 80% and 60% of the phenol is removed after 300 h. On the other hand, the KC and KCN carbons stand out, where equilibrium is achieved in about 100 h, and 94 and 98% of the phenol has been adsorbed. Figure 6 shows the results for KCN activated carbon. The plots of the other adsorbents are shown in Supplementary Materials (Figures S2–S6).

Generally, kenaf carbons activated with CO_2_ have a greater phenol adsorption capacity, while air-activated kenaf carbons (KAN and KAS) present poor results.

Various kinetic models (pseudo-first and pseudo-second order, Elovich, Natarajan-Khalaf and Bhattacharya-Venkobachar) have been applied to the experimental data obtained. The validity of the fit to one or the other model was evaluated by calculating the error with the following formula:

(1) $$Error=\frac{\sum_{1}^{n}\left(\left|q_{t,calc}-q_{t,exp}\right|\right)}{n}$$

where *q_t,calc_* is the adsorbed quantity calculated according to the model, *q_t,exp_* is the experimentally adsorbed quantity, and *n* is the number of data of the experiment. The results obtained are shown in Table 8.

Given the calculated error of kinetic results obtained, all the carbons seem to fit better to the pseudo-second order or Elovich kinetic models, especially at higher carbon concentrations. Although the errors obtained when applying the Elovich model are small, a better linear fit is obtained with the pseudo-second order model (Table S1). The fact that the Elovich and PSO models give good fits is not surprising, as both can be considered very similar when the degree of surface coating is low [50]. It also implies that the adsorption process developed experimentally by us is far from reaching saturation since the Elovich model is valid far from chemical equilibrium and the PSO model is valid when the ratio of occupied sites to the amount of adsorbate is high (see more information in the Supplementary Materials).

The model with the worst fit to the experimental data is that of Natarajan y Khalaf. The adjustment is more accurate the higher the proportion of adsorbent used. A slightly different behavior is KCN, whose kinetics do not fit the Elovich model particularly well but does fit the PSO model. It should be remembered that this is the activated carbon with which the best results have been obtained.

Table 9 shows the calculated values (PSO model) of k_2_ and adsorption capacity q_e_ for the 2 g L^−1^ test. For comparison, the experimental values of q_e_ are also shown. These are similar to the calculated values, which demonstrates the good fit of the PSO model. The rate constants and adsorption capacity are lower for the air-activated adsorbents than for the CO_2_-activated ones. In KA and KAS, this can be explained by a lower pore development, as shown in the characterization discussion. KAN has a higher specific surface area, but for some reason, the adsorption is slower (Figure S3). This can be explained by the pore width distribution not being adequate. Thus, this adsorbent has the smallest volume of macropores of all activated carbons and also stands out for having hardly any wide micropores, between 1 and 2 nm (Figure 3), which may hinder the diffusion of phenol.

It is more complex to explain why the two adsorbents with the largest BET surface area do not adsorb the most. One factor explaining this may be surface chemistry, which is more suitable for KCN than KCS (see the Section 2.4).

#### 2.3.2. Mechanistic Models

The adsorption mechanism is presumed to involve three stages that can influence the speed of the process: mass transfer of the adsorbate molecules across the external boundary layer (film diffusion), intraparticle diffusion, and diffusion into pores. One last step (adsorption at a site on the surface) is considered a fast process if it is physisorption (see below for theoretical simulations) and does not affect the adsorption kinetics.

Thus, diffusional kinetic models have also been applied to the adsorption of phenol to understand better the importance of the different stages in the adsorption process. We have therefore applied three mechanistic models: (a) the liquid film diffusion or Boyd model to study whether the determining step in the process is the mass transfer from solution; (b) the intra-particle diffusion model or Weber and Morris model; and (c) the Bangham model, which determines whether the limiting step is diffusion in narrow pores. The errors calculated from applying these three models to the experimental data are shown in Table 10. Also, since in order to consider one of the stages as limiting the process, a linearity in its fit must be observed, the values of R^2^ have been calculated (Table S2).

If we consider the calculated errors (Table 10), the best fits are achieved for the Bangham model, implying that the process’s rate-controlling step is diffusion inside the pores [51]. However, if there are several successive stages in a process, it can be perceived that some of the previous stages influence the speed since, before reaching the diffusion inside the pores, the molecules must be transferred from the liquid to the activated carbon and then enter through the wider pores until they reach the narrower pores. Therefore, in some cases, low error values are observed in the liquid film diffusion (KAN and KAS) or intraparticle diffusion (KAN) stages. It should be remembered that KAN carbon shows a somewhat different behavior compared to CO_2_ activated carbon, which has a similar specific surface area. This may be because there are several slow stages rather than a single one, which explains its slower adsorption.

As in the kinetic models, better fits are obtained using larger amounts of adsorbent. The amount of activated carbon used does not generally influence the adsorption mechanism.

If the R^2^ values are used, the conclusions are the same. Again, the best fit is achieved with the Bangham model, and for the KAN adsorbent, high R^2^ values are obtained for all three models.

### 2.4. DFT Simulations

**Figure 7:**
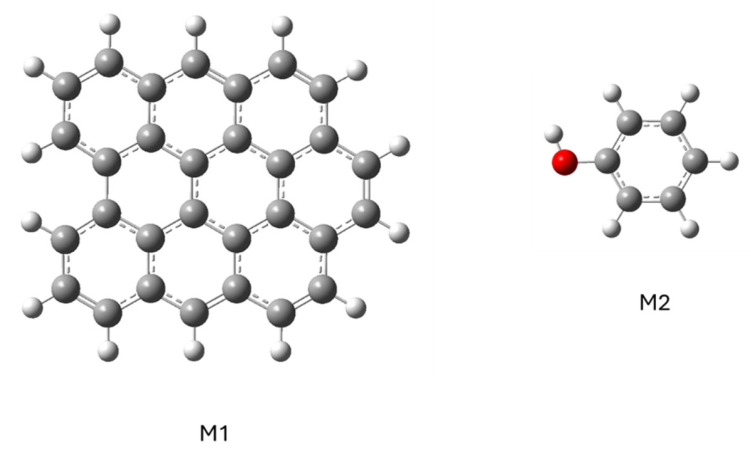
Representation of the structure of models of graphene (M1) and phenol (M2) molecules.

**Figure 8:**
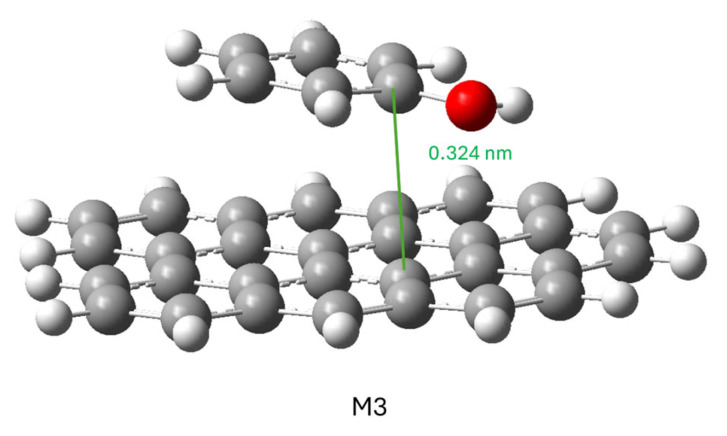
Representation of structure with minimal potential energy.

To study how a phenol molecule adsorbs on a graphene molecule, the M1 and M2 models were simulated separately (Figure 7) and then together (Figure 8).

To calculate the conjugate model, the phenol molecule was placed in various positions and orientations with respect to the graphene molecule: on the graphene molecule or to one side; with the hydroxyl group close to the graphene molecule, far away or in a neutral position; and with the phenol aromatic ring parallel or perpendicular to the plane of the graphene molecule. In all cases, the minimization of the ensemble’s energy led to the same result shown in Figure 8: the phenol molecule was positioned parallel to the graphene molecule, so this structure can be considered the most stable for this adsorption.

The interaction between the two gave a result of ΔG= −2.97 KJ mol^−1^. This differs from the situation in which both molecules are solvated in water, so the adsorption from the aqueous phase must be considered favorable.

The following calculated model was an attempt to study the energy of the phenol molecule’s approach to the graphene surface. The keyword scan was used to calculate the system’s energy as the two molecules gradually moved away from each other. Two close atoms, one from each molecule, were chosen and separated in 0.5 Å steps.

This simulation could not be completed because, after several steps, the calculation was aborted due to a problem related to the geometrical constraints imposed. However, in the few calculated steps, we could observe that the molecules did not separate from each other but that the phenol molecule slid over the surface of the graphene molecule to move the two atoms considered away from each other. This indicates that (a) the adsorption is stable; (b) the mobility of a phenol molecule on the surface of a carbonaceous material of this type is possible because no large variations in the energy of the system were observed; (c) the type of adsorption is physisorption.

**Figure 9:**
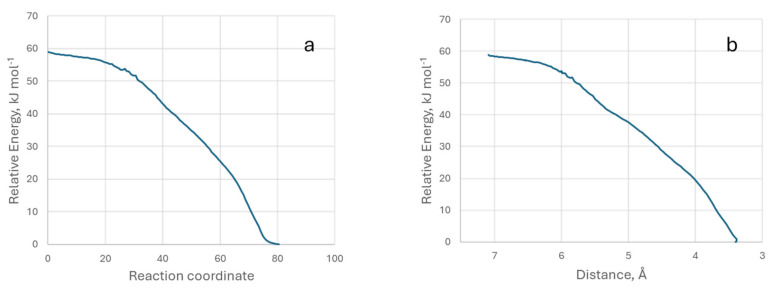
Representation of relative energy with respect to the reaction coordinate (**a**) and with respect to the distance between the two atoms (**b**). Model M3.

To carry out this study, we changed the method and used an IRC (intrinsic reaction coordinate) calculation [52]. This was possible, and Figure 9 shows the relative energy with respect to the reaction coordinate and the distance (0 equals the minimum energy distance) between the two reference atoms.

It can be seen that the energy decreases continuously as the two molecules approach each other, which corresponds to a typical physisorption process, and there is no activation energy in the adsorption. We have also provided a video (IRC-G.mp4) of the process from two perspectives.

**Figure 10:**
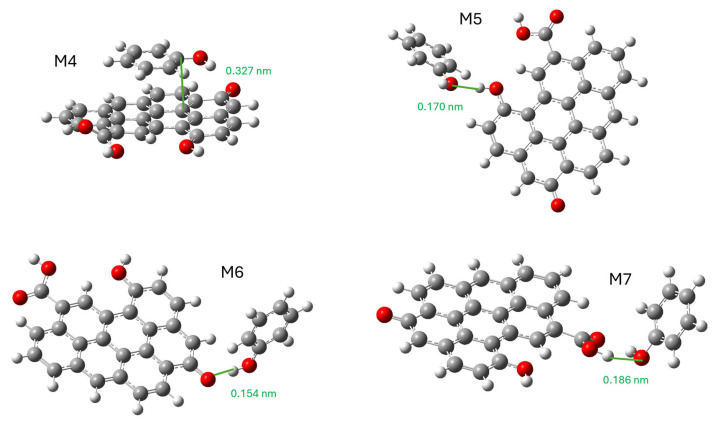
Stable structures of the interaction of phenol molecules with oxidized graphene. M4, π-π stacking; M5, interaction with hydroxyl; M6, interaction with carbonyl; and M7, interaction with carboxyl.

The following simulation was used to study the effect of oxygenated functional groups on adsorption. For this purpose, the M1 model was modified by adding a carboxyl, a carbonyl, and a hydroxyl group. The model phenol molecule was placed in such a way that it interacted with the graphene plane (π-π stacking) and each functional group. The result of the energy optimization is the four models (M4 to M7) shown in Figure 10. We have also provided an IRC video of the M4 model (IRC-GO.mp4).

The calculated ΔG, ΔH, and ΔS values are shown in Table 11.

These results show that binding by π-π stacking is more favorable if oxygenated groups are present (M4) than if they are absent (M3) for phenol adsorption. This result, which has traditionally been discussed in the literature, can be explained in this case for several reasons. One is that the oxidized graphene model has a high amount of oxygen (16.5 wt.%), implying a high degree of polarity. The second reason is that phenol has a polar functional group (-OH) and an apolar functional group (aromatic ring). Being a moderately polar substance, oxidized graphene can compete with the water solvation of phenol, increasing its adsorption capacity. In our case (Table 9), it can be observed that in the KA series the adsorption capacity decreases upon CA oxidation while in the KC series the opposite happens. It is a subject, therefore, where it is difficult to reach simple conclusions.

The adsorption enthalpy values are decisive since the best-performing models (M3, M4, and M6) clearly show more negative values than the other models. The presence of oxygenated groups represents a slight variation (−0.81 kJ mol^−1^) compared to their absence. The entropy variation presents two different behaviors. When phenol is directly bound to oxygenated groups (M5, M6, and M7), the entropy decreases less than in the other two models, so this thermodynamic factor is favored in these cases. At equal phenol adsorption position, the presence of oxygenated groups (M4) is slightly more favorable than their absence (M3).

It is also shown that the interaction of phenol with the different functional groups does not give the same results. While the interaction with a carbonyl group (M6) is even more favorable than π-π stacking, the interaction with hydroxyl or carboxyl is unfavorable. It should not be forgotten that this comparison is with respect to both model molecules being dissolved in water. Even a favorable interaction in absolute terms can give a negative result if the solvation of oxidized graphene and phenol separately is more stable. In this case, it is possible that the hydrogen-bridging bonds that can form between the hydroxyl and carboxyl groups with water explain this result. It is more difficult in carbonyl because it does not have hydrogen atoms bonded to electronegative atoms, so the loss of solvation with water is not so significant.

**Figure 11:**
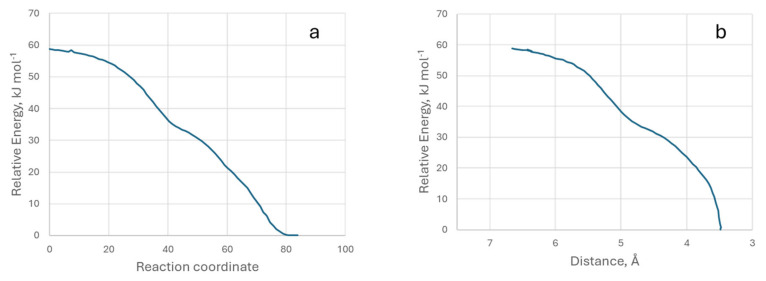
Representation of relative energy with respect to the reaction coordinate (**a**) and the distance between the two atoms (**b**). Model M4.

The IRC energy data for the formation of the M4 model are shown in Figure 11. We have also provided a video of the M4 model’s IRC.

Table 12 shows the calculated values for the HOMO and LUMO orbitals of the different systems, as well as the gap between them.

The HOMO energy values are similar to each other except for phenol. In the case of LUMO, the presence of oxygen atoms on graphene results in lower (more negative) energy values. The consequence of this fact is that the gap is smaller when the graphene is oxidized.

Using the NBO Analysis and the second-order perturbation theory, it is possible to calculate the donor–acceptor interactions between both molecules (phenol and oxidized graphene/graphene) and estimate the energetic importance of each interaction. Table 13 shows the main interactions for each calculated model. To help understanding, images of these models with the numbering of each atom are shown in Supplementary Materials (Figures S9–S13).

In the M3 model, an electron donation from the hydroxyl (O56 and H57) to an anti-bonding orbital of the C10 of graphene is observed. However, there is also a donation from the C9–C10 bond of graphene to the 1 s anti-bonding orbital of the H of hydroxyl.

In the M4 model the behavior is different: there are only donors on the oxidized graphene and acceptors on the phenol molecule. In all cases except the last one, they are π-π transfers and in the last one the acceptor is the H55 of the phenol hydroxyl group.

In the following models, the donor–acceptor interactions have a higher energetic value. This is due to the approaching polar functional groups that will give rise to dipoles and with them stronger van der Waals forces and even hydrogen bridge bonding. Thus, in the M5 model, the H37 of the hydroxyl of the oxidized graphene yields electrons to the C45–O54 bond of the phenol and to the H55 of the hydroxyl of the phenol, this being the most important donor–acceptor interaction. On the other hand, O54 of the phenol hydroxyl donates electrons to the H37 mentioned above.

In the M6 model, there is donation and acceptance in both directions, but the most important is from O35 carbonyl to H55 phenol hydroxyl. In this case, it can be stated that there is an evident hydrogen bridge bond. In the M7 model, donation acceptance is present in both directions as well. The most important is from H34 of carboxyl to H55 of phenol hydroxyl. The other two interactions can be considered a hydrogen bridge bond.

## 3. Materials and Methods

### 3.1. Preparation of the Samples

Activated carbons were prepared from kenaf using the inside of the stem. A vertical tubular furnace was used to produce biochar and activated carbons. The carbonization of the samples was carried out at 600 °C. The heating rate was 10 °C min^−1^ under a constant N_2_ flow of 200 mL min^−1^. The isothermal carbonization time was 2 h. The carbonized sample kenaf was named K0. The yield was 29.2%.

The activation temperatures were 750 °C when the activating agent was CO_2_ and 600 °C when the activating agent was air. In both cases, an activating agent flow equal to 200 mL min^−1^ was used, with a heating rate of 10 °C min^−1^. The isothermal activation time at maximum temperature was 2 h. The activated carbons were called KC and KA when the activating agents were CO_2_ and air, respectively. The yields were 56.1% (KA) and 78.4% (KC).

The acidic activated carbons were prepared by treating the activated carbons of kenaf with sulfuric (commercial 98%) or nitric acid (5 M) at a proportion of 0.05 g of carbon per milliliter of the acid used. The mixture was stirred for 90 min at ambient temperature. Later, the acidic carbons were filtered and washed with water in Soxhlet until a constant pH was reached, and then they were dried for 24 h at 110 °C.

### 3.2. Materials Characterization

Nitrogen adsorption isotherms at 77 K were determined using an Autosorb of Quantachrome (Anton Paar Spain S.L.U., Madrid, Spain) after outgassing the samples for 24 h at 140 °C to a residual vacuum. A Quantachrome porosimeter, Poremaster-60 (Anton Paar Spain S.L.U., Madrid, Spain), was used to obtain the mercury intrusion curves with sensitivity from 950 nm to 3.6 nm. The sample morphology was observed using a scanning electron microscope Hitachi S-4800 II (Hitachi Europe Ltd., Slough, UK); the imaging was performed in the high vacuum mode at an accelerating voltage of 10 kV, using secondary electrons.

The proximate analysis (moisture, fixed carbon, volatile matter, ash) was determined by a thermo-gravimetric method using a Mettler model TGA/SDTA 851e (Mettler Toledo, Cornellá del Llobregat, Spain). Moisture was measured by heating from 45 to 105 °C in an N_2_ atmosphere (30 mL min^−1^) at a heating rate of 10 °C min^−1^, with an isothermal time of 7 min at 105 °C. To determine the volatile matter content, the sample was heated to 600 °C at a heating rate of 25 °C min^−1^ and to 900 °C at a heating rate of 35 °C min^−1^, and this temperature was maintained for 7 min. Finally, at 900 °C, the N_2_ atmosphere was changed to air, and the sample was kept burning for 27 min. The weight lost was the fixed carbon content of the sample; the residual remaining mass was the ash content.

The elemental analysis (C, H, N, S, O) was carried out using a LECO CHNS-932 elemental analyzer (LECO Corporation, St. Joseph, MI, USA). C, H, N, and S were analyzed, and the difference was assigned to ash (proximate analysis) and oxygen content. Moisture determined by thermogravimetry was deduced.

X-ray photoelectronic spectra were obtained with a K-Alpha de Thermo Scientific (Thermo Fisher Scientific, Waltham, MA, USA) photoelectron spectrometer using monochromatic Al-K_α_ radiation. The source operated at 12 kV, 6 mA. The measurements were performed under a vacuum of 2.2 × 10^−5^ Pa.

The points of zero charge (PZC) values were determined using the method proposed by Valente Nabais and Carrott modified [53]. The samples were placed in contact with a NaNO_3_ 0.1 M solution and stirred for 48 h at 25 °C. After this time, the sample was filtered, the pH of the solution was measured, and a PZC value was assigned to it.

Finally, the number of acidic and basic groups was evaluated by acid-base titration. To determine the acid groups, 0.15 g samples were suspended and stirred in a 30 mL solution of 0.01 M NaOH for 24 h at 25 °C, and the resulting solution was titrated with a 0.01 M HNO_3_ solution. Then, to determine alkaline groups, the activated carbons (0.15 g) were suspended in a 0.01 M HNO_3_ solution under the same conditions and titrated with a 0.01 M NaOH solution.

### 3.3. Adsorption

To study the adsorption kinetics of both adsorbates, a solution of 100 mg L^−1^ of phenol was used at pH = 7 and keeping the ionic strength constant at 0.01 M.

The kinetic study was carried out in glass tubes in which 25 mL of the starting solution and the corresponding amount of adsorbent were added in each case. The experiments were carried out at 25 °C in a thermostatic platform bath with a stirring of 45 min^−1^. The pH was achieved by adding phosphate salts (Na_2_HPO_4_/NaH_2_PO_4_).

The adsorbate concentration was determined after filtering the sample with a 0.45 µm filter using a Varian Cary 50 Probe UV-Visible spectrophotometer (Agilent, Santa Clara, CA, USA) at a wavelength of 269 nm (the wavelength of maximum phenol absorption). The tests were repeated at least twice. The phenol UV-Vis spectrum and calibration curve are shown in Figures S7 and S8.

### 3.4. Theoretical Model Fits

Further information on the kinetic and mechanistic models used in this research can be found in the Supplementary Materials.

No lineal correlation was used to evaluate the model’s goodness of fitting and suitability. The R^2^ values of the linear fit (Tables S1 and S2) have been included in the Supplementary Materials for comparison purposes.

Although the linear regression method is the most widely used due to its simplicity, it is known [54] that the linearization process changes the independent/dependent variables. Also, this process could introduce propagate errors to the independent/dependent variables, which could cause inaccurate estimations of the parameters. Models with more than two variables, such as Elovich’s, must assume certain approximations. The nonlinear method can provide consistent and accurate estimations for model parameters [55].

### 3.5. DFT Calculations

The computational study was performed using the Gaussian16 (Revision A.03) package [56] with the PW6B95D3/Def2SVP level of theory. The calculations were carried out in water (SMD model) [57]. Given the difficulty of calculating unbound systems and the fact that we initially observed that the changes in the system’s potential energy were very small and made it difficult to reach a minimum, we selected the option opt = tight for the optimization. A consequence of this decision is that more steps are needed to obtain the minimum energy. For the same reason, we use the option scf = (xqc,verytight).

The thermodynamic parameters for the two-molecule models were calculated by subtracting the calculated data of the individual molecules from the joint model result.

To study which are the most important interactions between phenol and graphene we have used NBO Analysis. This method transforms the wave functions used in the DFT calculation into localized forms (bonds and orbitals). It is often used to determine the Lewis structure of a compound but, in our case, we have used the second order perturbation theory to calculate the donor–acceptor interactions and estimate the energetic importance of each one.

## 4. Conclusions

Kenaf core is a material suitable for manufacturing activated carbons for use in the adsorption of phenolic compounds. The activation method and the subsequent modification of the chemical surface with mineral acids strongly influence the adsorption capacity. Very different products can be obtained from the same biochar, so knowing the preparation methods and their consequences is essential. The use of mineral acids increases the specific surface area, oxidizes the surface of the activated carbon depending on the acid used, and increases the adsorption capacity.

The kinetic studies carried out preferably follow the PSO and Elovich models. The determining step in the adsorption kinetics is the diffusion of phenol into the pores, although all steps seem to influence the process for an adsorbent.

Simulations of adsorption by the DFT method show that the presence of oxygenated groups in the adsorbent improves the adsorption capacity by π-π stacking. However, oxygenated functional groups can favor or disfavor the binding of phenol to them depending on the type of functional group.

The DFT method has been shown to be an important tool for understanding adsorption phenomena, so its use should be expanded in future studies. In addition to the results shown here, other factors that influence the adsorption process, such as pH, temperature, or adsorbate concentration, should be studied. The adsorbents used are likely to be used to remove other contaminants.

## Figures and Tables

**Table 1:** Used adsorbents.

Code	Precursor	Carbonization	Activation	Acid Treatment
K0	Kenaf	Yes	No	No
KA	K0	No	Air	No
KAN	KA	No	No	HNO_3_
KAS	KA	No	No	H_2_SO_4_
KC	K0	No	CO_2_	No
KCN	KC	No	No	HNO_3_
KCS	KC	No	No	H_2_SO_4_

**Table 2:** Textural properties of the carbonized and activated samples.

		D-R			t-Plot		
Sample	S_BET_ (m^2^ g^−1^)	W_0_ (cm^3^ g^−1^)	E_0_ (KJ mol^−1^)	L (nm)	W_0_ (cm^3^ g^−1^)	V_meso_ (cm^3^ g^−1^)	V_macro_ (cm^3^ g^−1^)
K0	29	0.017	13.7	0.95	0.013	0.047	3.664
KA	15	0.004	10.3	1.26	0.000	0.099	4.830
KAN	315	0.153	19.7	1.32	0.115	0.080	4.427
KAS	106	0.036	13.4	0.97	0.023	0.101	5.075
KC	124	0.035	14.7	0.89	0.020	0.034	6.229
KCN	185	0.084	19.4	0.67	0.043	0.045	4.520
KCS	316	0.137	19.0	0.68	0.124	0.085	5.414

V_meso_: Mesopores volume. V_macro_: Macropores volume.

**Table 3:** Elemental and proximate analysis.

Sample	Elemental Analysis (wt %)					Proximate Analysis (wt %)		
	C	H	N	S	O	VM	Ash	FC
K0	89.7	2.2	0.8	0.3	6.9	17.5	5.8	76.7
KA	91.0	1.8	0.7	0.4	6.1	15.2	6.5	78.3
KAN	80.4	1.9	1.8	0.2	15.8	27.4	2.0	70.6
KAS	87.9	2.1	0.8	0.8	8.3	22.2	6.5	71.3
KC	90.7	1.6	1.1	0.5	6.0	17.7	7.2	75.1
KCN	86.9	0.9	1.5	0.5	10.3	18.6	1.4	80.0
KCS	91.8	0.8	0.8	0.9	5.7	14.5	1.7	83.8

Elemental analysis as a percentage of the organic fraction on a wt.% dry biomass basis. Proximate analysis in terms of wt.% dry biomass basis. VM: volatile matter. Ash: mineral matter. FC: fixed carbon.

**Table 4:** Elemental analysis (XPS) composition.

Sample	Elemental Analysis (wt %)			
	C	O	N	S
K0	78.6	16.6	0.9	3.9
KA	85.7	12.9	0.7	0.6
KAN	83.1	14.6	2.0	0.3
KAS	86.7	11.4	1.0	1.0
KC	87.5	10.6	1.0	0.9
KCN	84.1	13.6	1.6	0.7
KCS	88.8	9.1	0.8	1.3

**Table 5:** The content of surface groups of carbons determined by the XPS method.

Concentration on the Surface (% At)					
Sample	-C-C (284.8)	C-O (285.8–286.4)	C=O (287.8–287.9)	COO (288.9–291.5)	π-π Transition (293.1–293.9)
K0	70.3	21.5	-	3.1	5.0
KA	69.1	22.3	-	7.5	1.0
KAN	58.9	19.9	-	21.2	-
KAS	53.6	21.4	-	24.9	-
KC	59.8	30.0	-	7.7	2.5
KCN	43.3	28.9	-	23.3	4.5
KCS	48.9	25.6	-	25.5	-

**Table 6:** The content of surface groups of nitrogen and sulfur determined by the XPS method.

Concentration on the Surface (% At)				
Sample	N_reduced_ (398.2–400.7)	N_oxidized_ (405.8–405.9)	S_reduced_ (162.8–165.2)	S_oxidized_ (168.5–170.6)
K0	100.0	-	5.6	94.4
KA	100.0	-	43.6	56.4
KAN	47.3	52.7	53.9	46.1
KAS	100.0	-	21.6	78.4
KC	100.0	-	93.3	6.7
KCN	69.3	30.7	92.2	7.8
KCS	100.0	-	47.9	52.1

**Table 7:** PZC and total acidity and basicity of the samples.

Sample	PZC	Total Acidity Meq H^+^ g^−1^	Total Basicity Meq OH^−^ g^−1^
K0	9.9	0.10	0.88
KA	10.3	0.06	1.40
KAN	6.3	0.45	0.36
KAS	6.2	0.32	0.34
KC	10.3	0.06	1.59
KCN	6.7	0.44	0.34
KCS	6.8	0.36	0.39

**Table 8:** Error values calculated from the application of the different models to kenaf carbons.

Model	Sample	Concentration of Carbon				
		0.1 g L^−1^	0.25 g L^−1^	0.5 g L^−1^	1 g L^−1^	2 g L^−1^
Pseudo-first order	KA	9.7	3.7	4.4	4.9	4.4
	KAN	7.5	4.4	2.0	1.6	1.0
	KAS	7.4	2.9	2.3	1.5	1.8
	KC	14.0	13.6	11.9	6.6	2.6
	KCN	12.8	7.3	5.8	4.7	2.5
	KCS	18.1	5.8	6.0	5.9	3.6
Pseudo-second order	KA	7.9	3.9	2.9	3.8	2.7
	KAN	8.3	5.4	1.9	1.6	0.9
	KAS	7.4	2.3	2.0	1.2	1.5
	KC	10.0	13.7	7.2	4.5	1.2
	KCN	19.9	5.9	4.5	4.8	1.8
	KCS	14.8	4.1	4.5	3.7	2.5
Elovich	KA	6.1	4.2	1.7	1.7	0.9
	KAN	7.5	5.4	4.6	2.9	0.8
	KAS	8.3	2.5	1.8	1.6	0.5
	KC	11.2	12.9	4.9	2.3	2.1
	KCN	24.4	5.6	4.0	6.4	4.1
	KCS	18.8	3.3	3.4	1.8	0.9
Natarajan and Khalaf	KA	35.1	25.9	21.0	13.9	7.3
	KAN	13.5	6.3	2.7	3.2	2.9
	KAS	16.3	9.9	8.8	8.1	5.9
	KC	55.8	36.2	31.0	16.0	4.0
	KCN	34.4	28.5	24.9	9.0	1.6
	KCS	38.1	32.4	28.4	18.1	6.3
Bhattacharya and Venkobachar	KA	11.4	3.5	4.8	4.9	4.1
	KAN	9.6	5.7	1.6	1.7	0.8
	KAS	8.3	2.6	2.3	2.0	1.9
	KC	13.5	19.1	9.9	6.2	2.7
	KCN	12.8	5.9	7.6	4.7	2.2
	KCS	24.6	5.9	6.8	7.0	3.6

**Table 9:** Pseudo-second order constant for the adsorption of phenol in activated carbons (rate: 2 g L^−1^).

Sample			Experimental q_e_, mg g^−1^
KA	q_e_, mg g^−1^	43.48	42.68
	k_2_, g h^−1^ mg^−1^·10^−4^	7.1	
KAN	q_e_, mg g^−1^	25.85	23.62
	k_2_, g h^−1^ mg^−1^·10^−4^	3.0	
KAS	q_e_, mg g^−1^	35.19	34.27
	k_2_, g h^−1^ mg^−1^·10^−4^	4.9	
KC	q_e_, mg g^−1^	48.73	48.64
	k_2_, g h^−1^ mg^−1^·10^−4^	37.9	
KCN	q_e_, mg g^−1^	51.13	48.80
	k_2_, g h^−1^ mg^−1^·10^−4^	14.8	
KCS	q_e_, mg g^−1^	43.64	46.18
	k_2_, g h^−1^ mg^−1^·10^−4^	67.4	

**Table 10:** Error values from the application of the different models to carbons.

Model	Sample	Concentration of Carbon				
		0.1 g L^−1^	0.25 g L^−1^	0.5 g L^−1^	1 g L^−1^	2 g L^−1^
Bangham	KA	7.1	5.2	2.8	1.6	0.8
	KAN	7.6	4.3	1.3	0.9	0.6
	KAS	7.1	2.6	2.0	1.9	0.5
	KC	12.7	11.0	5.4	2.7	1.0
	KCN	17.0	10.3	5.6	4.3	1.2
	KCS	15.8	4.8	4.9	1.9	1.0
Intraparticle Diffusion	KA	13.5	13.3	10.8	9.0	6.8
	KAN	8.8	6.0	1.7	0.7	0.6
	KAS	9.5	3.7	3.8	4.4	3.6
	KC	27.3	17.4	20.2	16.5	13.5
	KCN	19.8	17.0	17.0	16.8	12.6
	KCS	24.9	20.1	17.9	14.5	9.5
Liquid film diffusion	KA	10.7	5.2	4.5	4.9	4.1
	KAN	7.3	4.7	2.3	1.8	1.0
	KAS	11.4	5.4	2.3	2.0	1.8
	KC	15.1	13.4	9.9	7.2	2.6
	KCN	23.2	7.2	7.6	4.7	2.2
	KCS	17.2	6.2	6.4	5.9	3.6

**Table 11:** Calculated ΔG values for the different simulations.

Model	ΔG, kJ mol^−1^	ΔH, kJ mol^−1^	ΔS, J mol^−1^ K^−1^
M3	−2.97	−55.02	−174.59
M4	−4.67	−55.83	−171.60
M5	11.93	−30.73	−143.07
M6	−11.47	−58.21	−156.75
M7	15.91	−30.55	−155.82

**Table 12:** Calculated energy values (in eV) for HOMO and LUMO orbitals.

Model	E_HOMO_	E_LUMO_	Gap
M1 (grahpene)	−5.21	−2.42	2.79
M2 (phenol)	−6.54	−0.03	6.52
Oxidized graphene	−5.16	−3.56	1.60
M3	−5.19	−2.40	2.78
M4	−5.14	−3.54	1.60
M5	−5.42	−3.62	1.81
M6	−5.12	−3.51	1.61
M7	−5.17	−3.58	1.59

**Table 13:** Main donor–acceptor interactions in M3, M4, M5, M6, and M7 models.

Model	Donor NBO	Acceptor NBO	E (kJ mol^−1^)
M3	BD (2) C 9–C 10	LP*(1) H 57	3.10
	LP (3) O 56	RY*(3) C 10	2.05
	LP*(1) H 57	RY*(3) C 10	3.60
M4	BD (2) C 4–C 5	BD*(2) C 44–C 45	1.88
	BD*(2) C 14–C 15	BD*(2) C 43–C 48	2.93
	BD*(2) C 14–C 15	BD*(2) C 44–C 45	3.35
	BD*(2) C 17–C 19	BD*(2) C 43–C 48	2.76
	BD (2) C 9–C 40	LP*(1) H 55	2.64
M5	LP*(1) H 37	BD*(1) C 45–O 54	13.01
	LP*(1) H 37	LP*(1) H 55	296.23
	LP (1) O 54	LP*(1) H 37	92.47
	LP (2) O 54	LP*(1) H 37	40.88
M6	BD*(2) C 9–C 40	BD*(2) C 43–C 44	8.70
	LP (2) O 35	LP*(1) H 55	282.29
	LP (1) O 35	LP*(1) H 55	32.34
	LP*(1) H 55	BD*(1) C 3–O 35	10.79
M7	LP (2) O 32	LP*(1) H 55	59.58
	LP*(1) H 34	LP*(1) H 55	284.09
	LP (2) O 54	LP*(1) H 34	63.60

BD: bond; LP: lone pair; RY: Rydberg orbital; *: anti-bonding orbital.

## Data Availability

Dataset available on request from the authors.

## Supplementary Materials

The following supporting information can be downloaded at: https://www.mdpi.com/article/10.3390/molecules29204941/s1, Figure S1. SEM images of samples (a) KAN; (b) KAS; (c) KCN; and (d) KCS; Figure S2. Phenol adsorption kinetics with KA activated carbon using different proportions of adsorbent; Figure S3. Phenol adsorption kinetics with KAN activated carbon using different proportions of adsorbent; Figure S4. Phenol adsorption kinetics with KAS activated carbon using different proportions of adsorbent; Figure S5. Phenol adsorption kinetics with KC activated carbon using different proportions of adsorbent; Figure S6. Phenol adsorption kinetics with KCS activated carbon using different proportions of adsorbent; Figure S7. UV spectrum of phenol; Figure S8. Calibration curve of phenol solution; Figure S9. Numbering of atoms in the M3 model; Figure S10. Numbering of atoms in the M4 model; Figure S11. Numbering of atoms in the M5 model; Figure S12. Numbering of atoms in the M6 model; Figure S13. Numbering of atoms in the M7 model. Table S1. R^2^ values of the application of the pseudo-second order and Elovich kinetic models to the experimental data; Table S2. R^2^ values of the application of Bangham, intraparticle diffusion and film diffusion models to the experimental data; information about theoretical kinetic models used in this research with references. References [58,59,60,61,62,63,64,65,66] are cited in the Supplementary Materials.
