# Supplementary material for: Kinetic and Mechanistic Analysis of Phenol Adsorption on Activated Carbons from Kenaf
Source: Molecules. 2024 Oct 18;29(20):4941. doi: 10.3390/molecules29204941 (PMC11510434; doi:10.3390/molecules29204941)
Supplement: Supplementary file 1 [file molecules-29-04941-s001.zip › molecules-3220263-supplementary.pdf]

Supplementary Material

Kinetic and Mechanistic Analysis of Phenol Adsorption on Activated Carbons from Kenaf

Delia Omenat-Morán, Carlos J. Durán-Valle, Manuel A. Martínez-Cañas

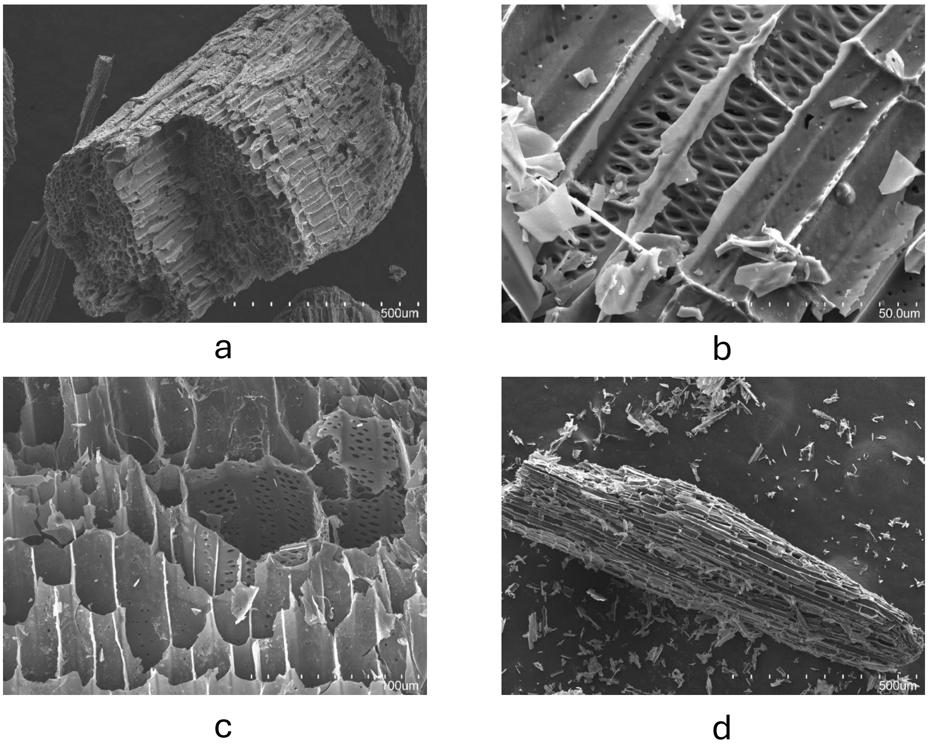

Figure S1. SEM images of samples a) KAN; b) KAS; c) KCN; and d) KCS.

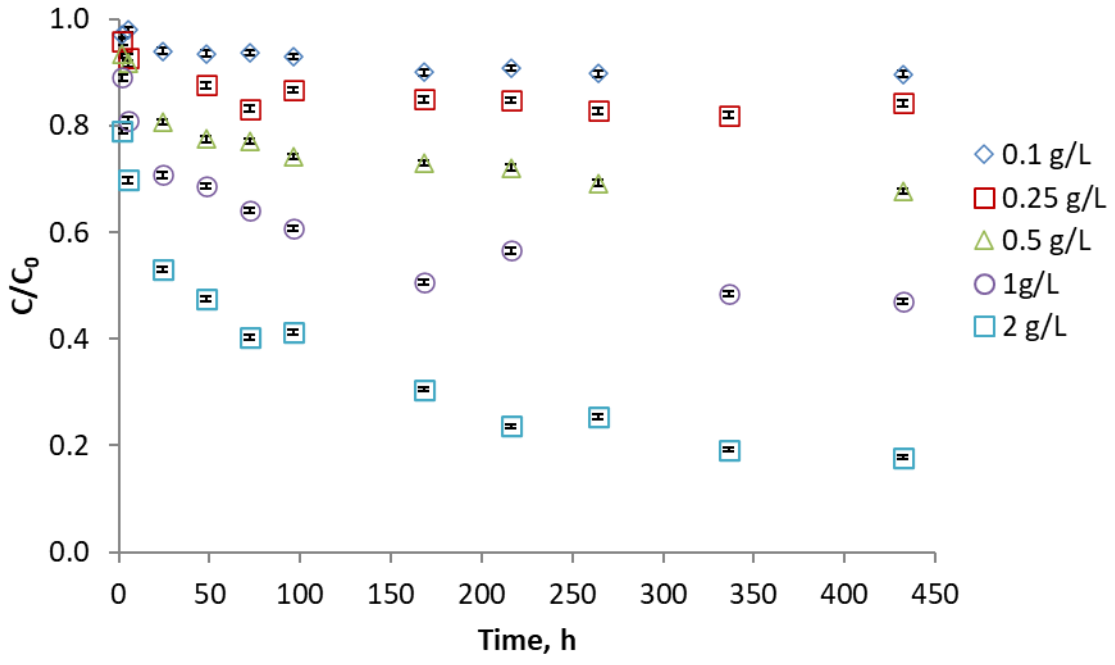

Figure S2. Phenol adsorption kinetics with KA activated carbon using different proportions of adsorbent.

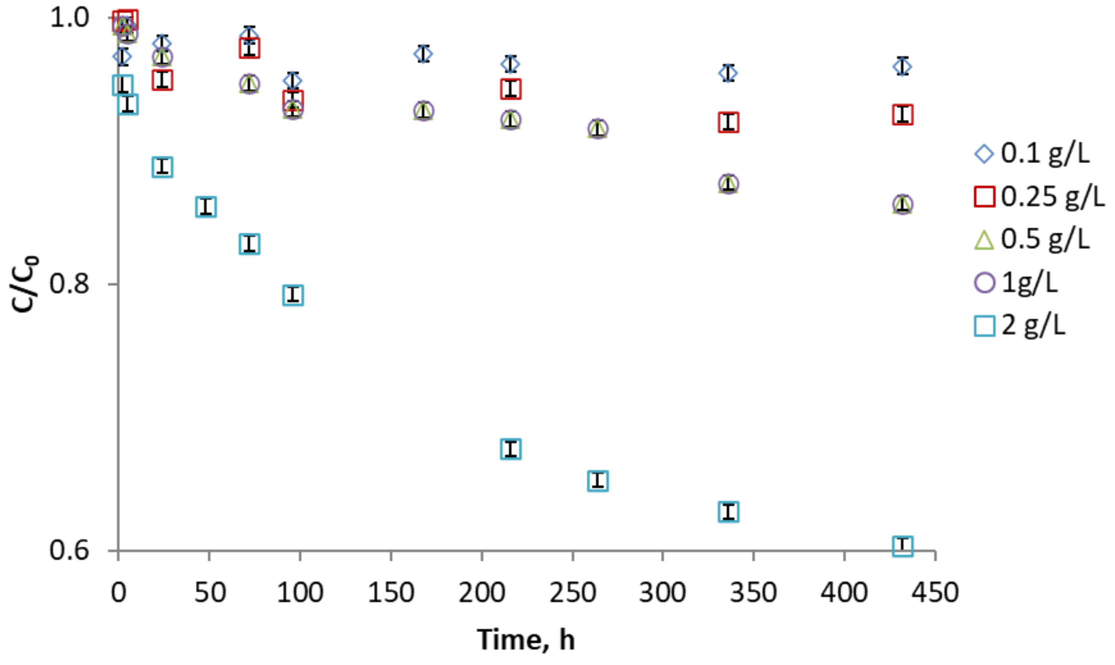

Figure S3. Phenol adsorption kinetics with KAN activated carbon using different proportions of adsorbent.

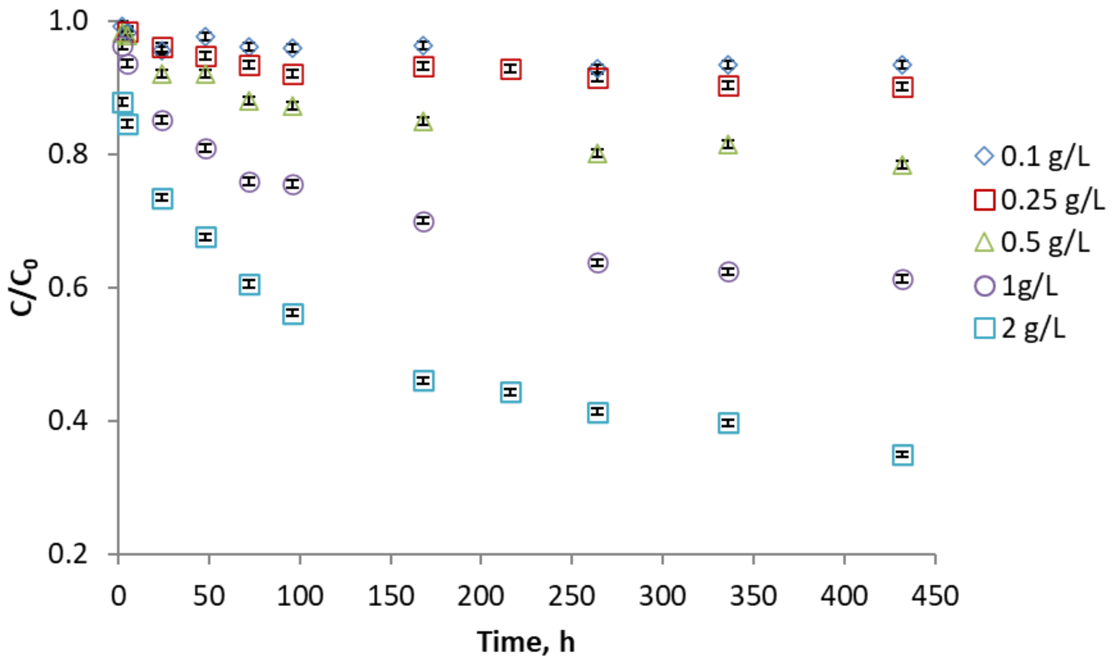

Figure S4. Phenol adsorption kinetics with KAS activated carbon using different proportions of adsorbent.

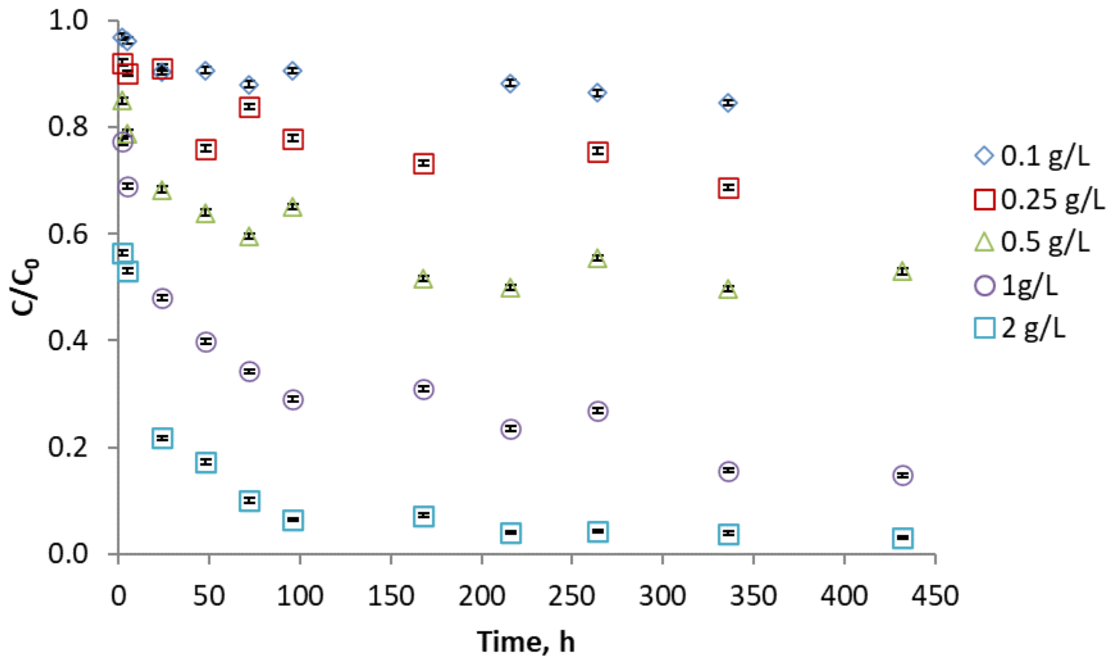

Figure S5. Phenol adsorption kinetics with KC activated carbon using different proportions of adsorbent.

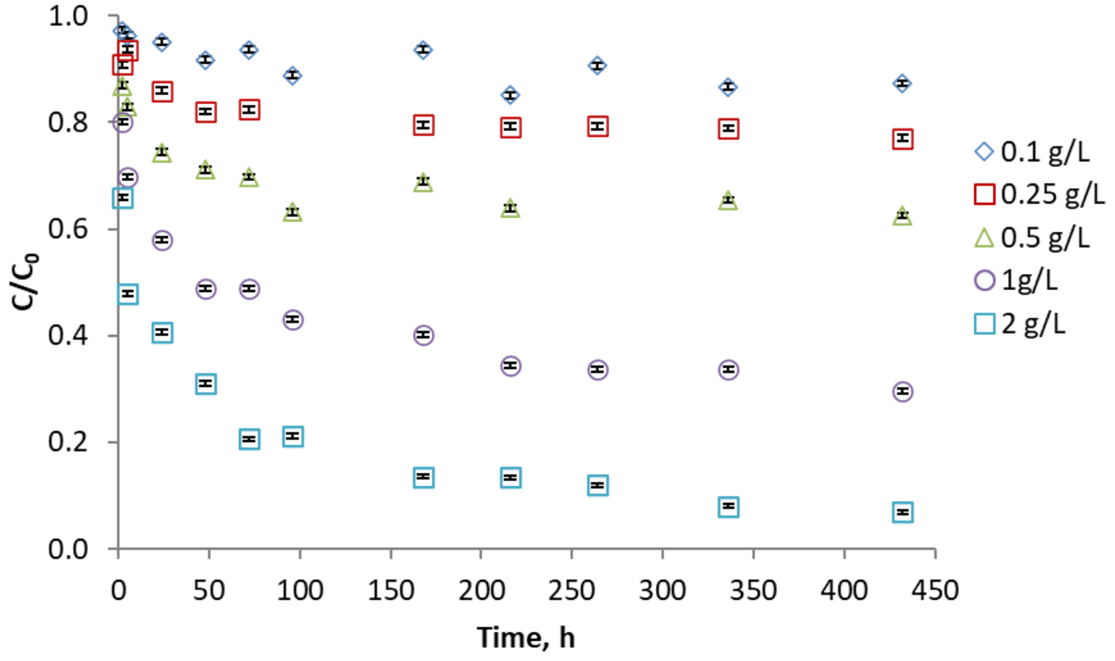

Figure S6. Phenol adsorption kinetics with KCS activated carbon using different proportions of adsorbent.

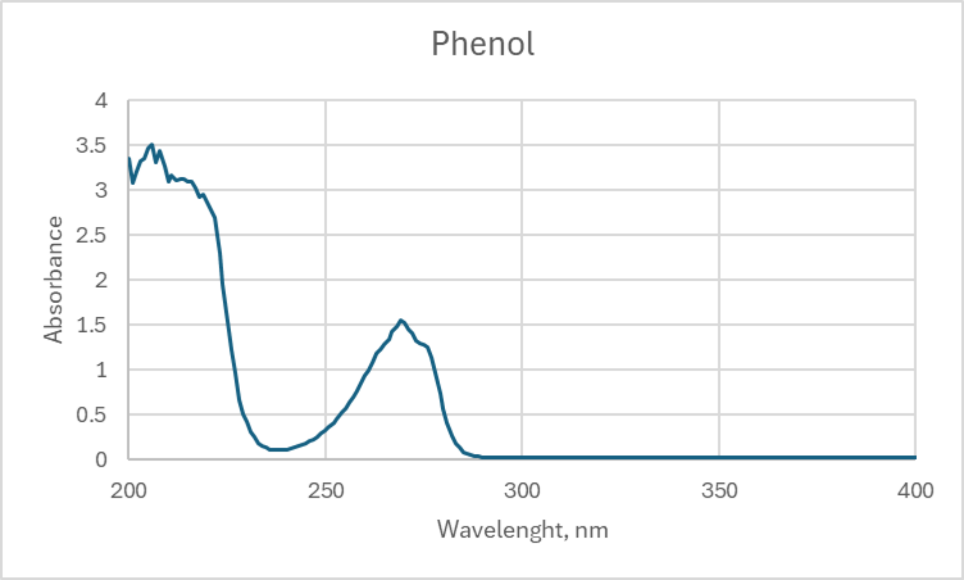

Figure S7. UV spectrum of phenol.

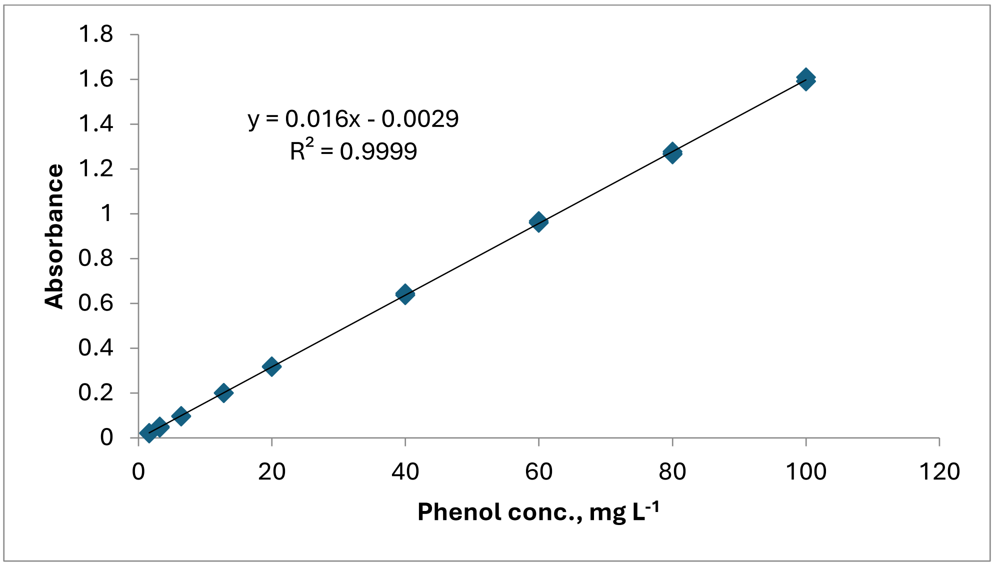

Figure S8. Calibration curve of phenol solution.

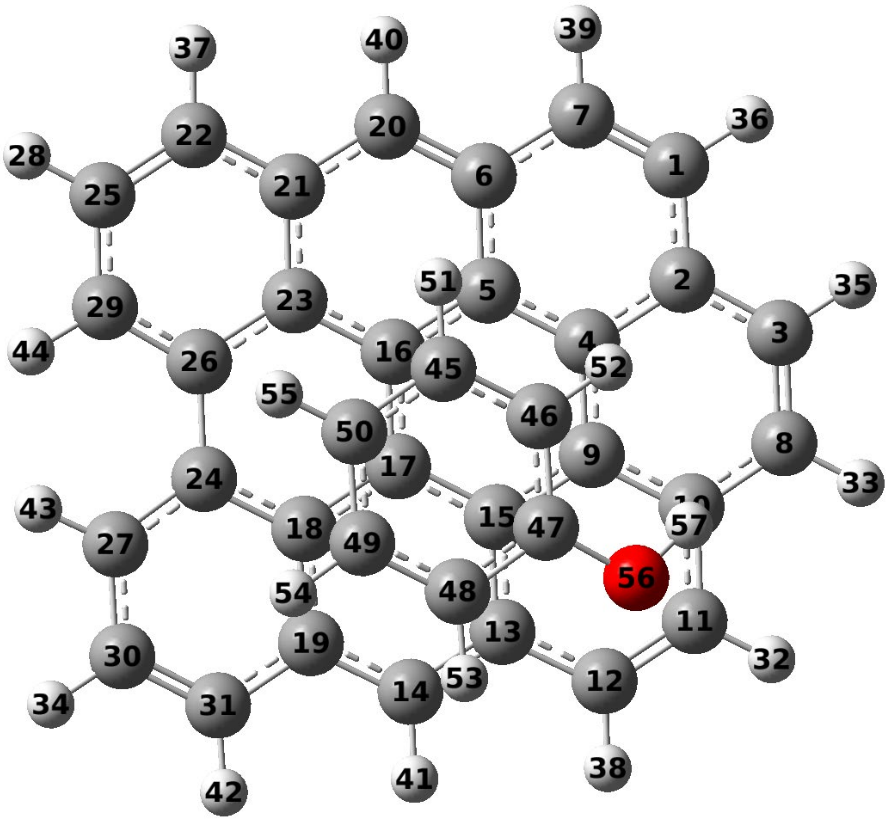

Figure S9. Numbering of atoms in the M3 model.

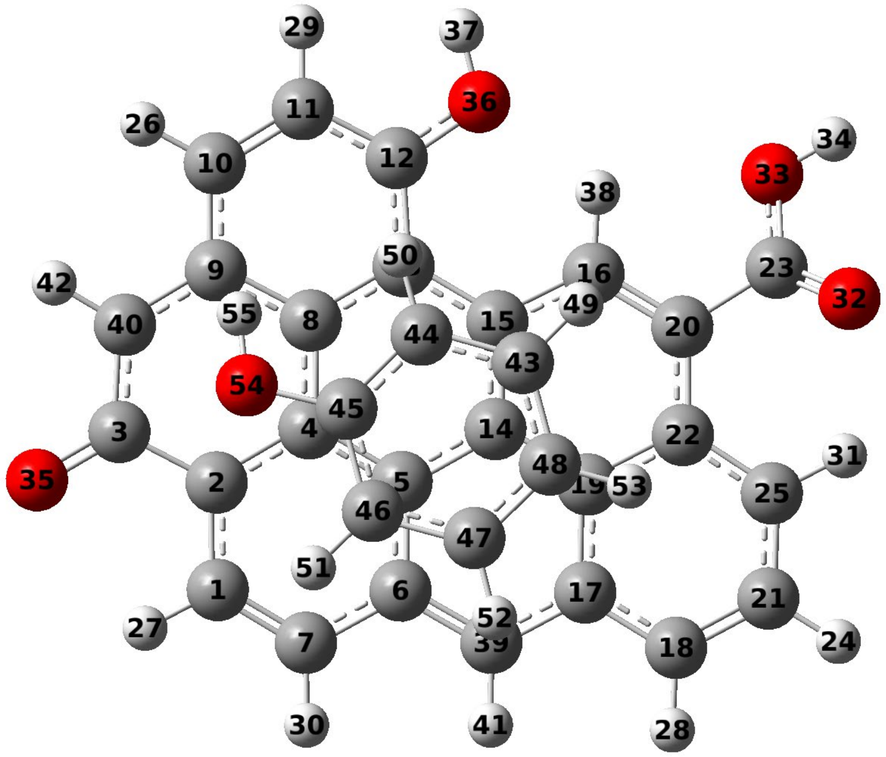

Figure S10. Numbering of atoms in the M4 model.

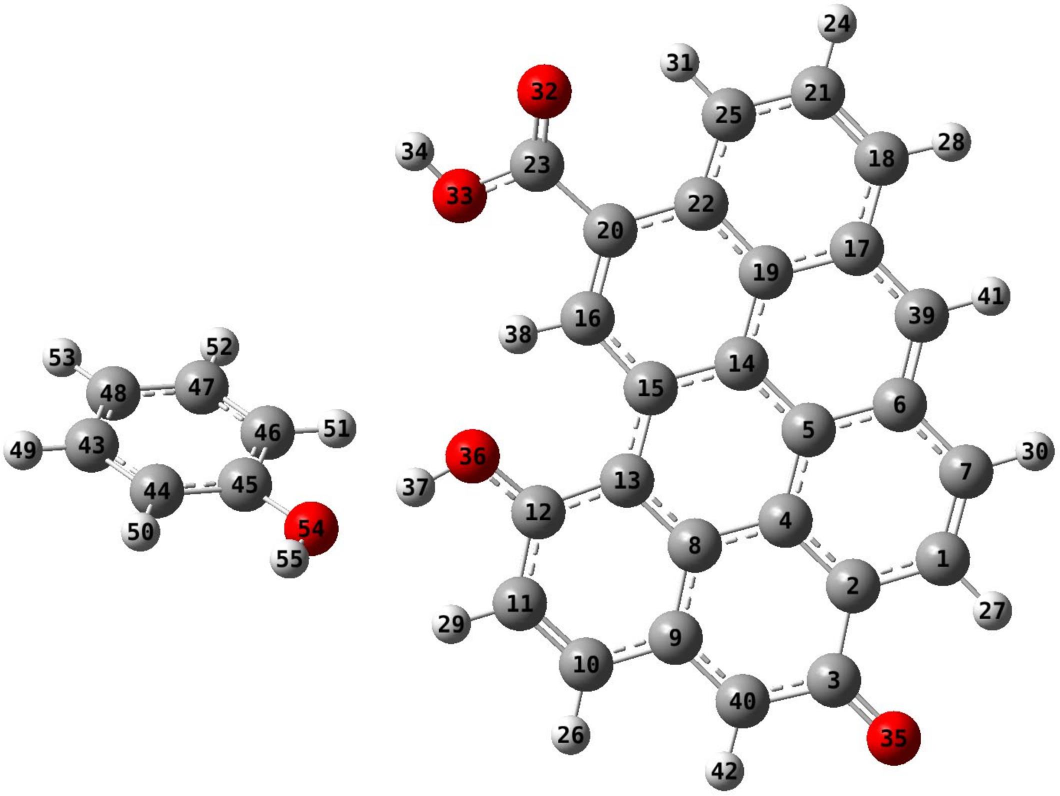

Figure S11. Numbering of atoms in the M5 model.

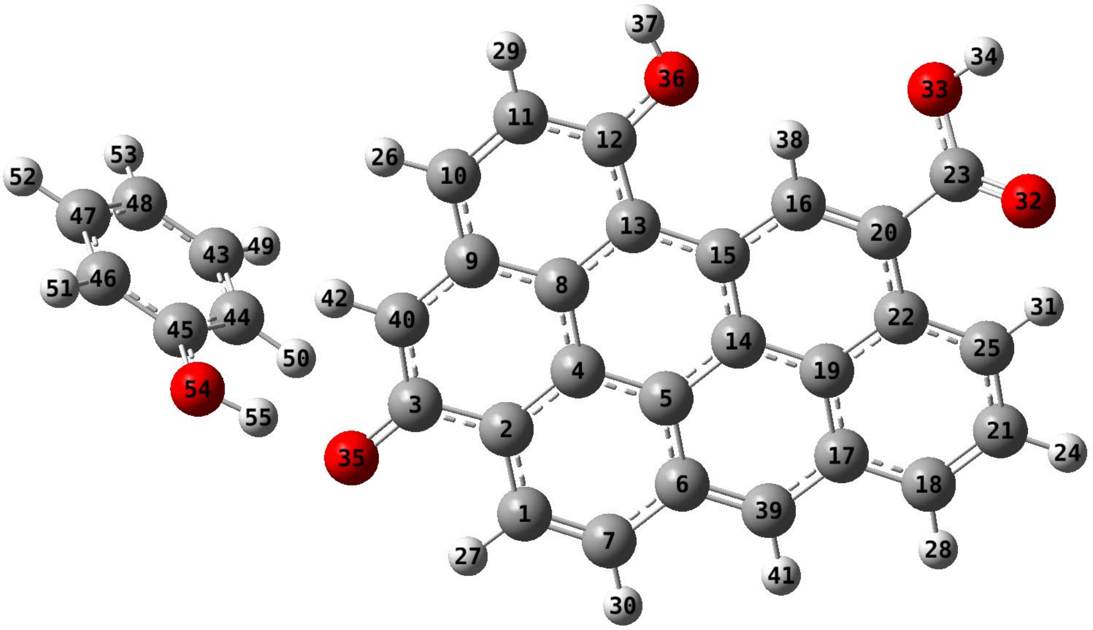

Figure S12. Numbering of atoms in the M6 model.

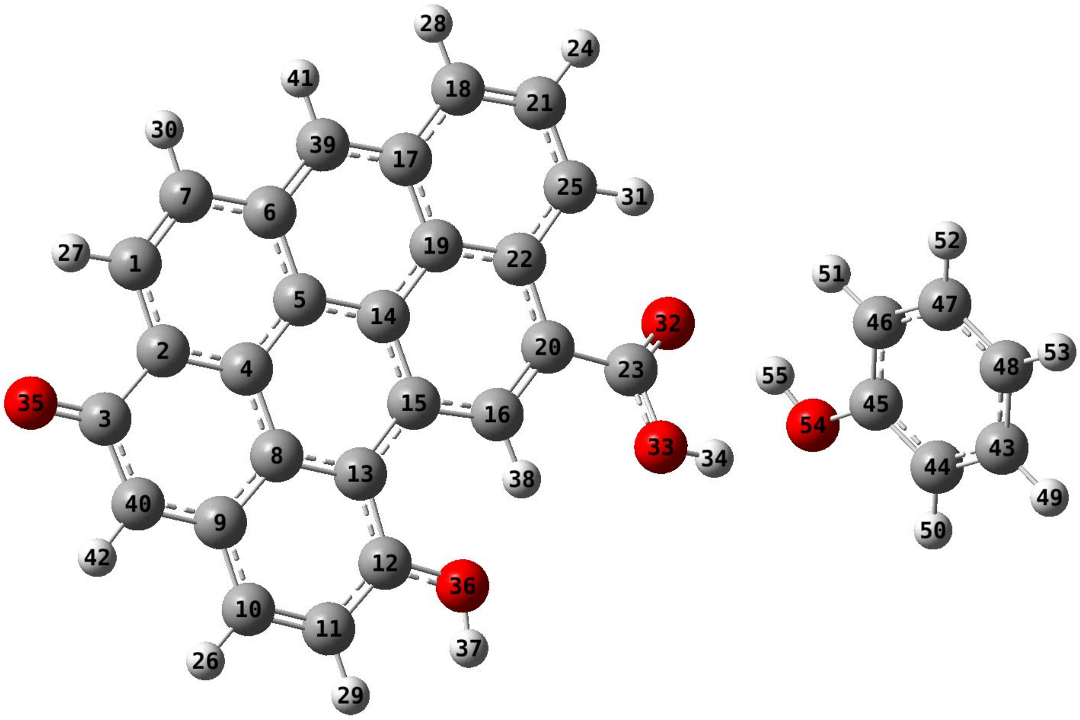

Figure S13. Numbering of atoms in the M7 model.

Table S1. R^2 values of the application of the pseudo-second order and Elovich kinetic models to the experimental data.

Model	Sample	Concentration of adsorbent				
		0.1 g L^{-1}	0.25 g L^{-1}	0.5 g L^{-1}	1 g L^{-1}	2 g L^{-1}
Pseudo-first order	KA	0.991	0.993	0.996	0.990	0.994
	KAN	0.879	0.775	0.854	0.906	0.949
	KAS	0.920	0.950	0.985	0.994	0.988
	KC	0.987	0.980	0.991	0.994	1.000
	KCN	0.881	0.981	0.993	0.996	0.999
	KCS	0.914	0.994	0.994	0.997	0.998
Elovich	KA	0.904	0.878	0.986	0.970	0.986
	KAN	0.318	0.790	0.826	0.849	0.867
	KAS	0.766	0.916	0.944	0.972	0.957
	KC	0.910	0.808	0.942	0.979	0.921
	KCN	0.712	0.939	0.966	0.923	0.900
	KCS	0.706	0.901	0.923	0.981	0.976

Table S2. R^2 values of the application of Bangham, intraparticle diffusion and film diffusion models to the experimental data.

Model	Sample	Concentration of adsorbent				
		0.1 g L^{-1}	0.25 g L^{-1}	0.5 g L^{-1}	1 g L^{-1}	2 g L^{-1}
Bangham	KA	0.869	0.878	0.949	0.973	0.992
	KAN	0.319	0.817	0.988	0.979	0.980
	KAS	0.838	0.923	0.970	0.982	0.995
	KC	0.902	0.826	0.943	0.975	0.967
	KCN	0.887	0.856	0.927	0.944	0.925
	KCS	0.827	0.845	0.930	0.970	0.976
Intraparticle diffusion	KA	0.615	0.074	0.352	0.462	0.343
	KAN	0.488	0.784	0.960	0.985	0.990
	KAS	0.796	0.777	0.909	0.858	0.811
	KC	0.253	0.388	-0.060	-0.010	-2.040
	KCN	0.801	0.762	0.755	0.540	0.101
	KCS	0.452	-0.36	-0.760	-0.270	-0.850
Film diffusion	KA	0.652	0.540	0.839	0.894	0.920
	KAN	0.412	0.514	0.795	0.898	0.980
	KAS	0.872	0.712	0.954	0.985	0.904
	KC	0.239	0.324	0.413	0.634	0.647
	KCN	0.694	0.972	0.946	0.639	0.756
	KCS	0.528	0.826	0.301	0.860	0.712

Information about theoretical kinetic models used in this research

In the present work, the pseudo-first-order kinetic model, pseudo-second-order model, Elovich equation, Natarajan and Khalaf model and Bhattacharya and Venkobachar model were used to investigate the adsorption of phenol.

In addition, diffusion models have been studied to describe the process of film diffusion and intraparticle diffusion. Bangham model, intraparticle diffusion kinetic model and diffusion model in kinetic liquid film were used to investigate this process.

The kinetics studies were performed at 25 º C, and solution pH was 7 and ionic force was adjusted at 0.01 M. The initial concentration of phenol was set at 100 mg L^{-1}, and the samples were separated at predetermined time intervals. The variable studied in all of them has been the concentration of carbon so that the concentrations 0.1, 0.25, 0.5, 1 and 2 g L^{-1} have been studied.

The pseudo-first-order kinetic model [58] is expressed as follows (Equation S1): where k_1 (h^{-1}) is the first-order rate constant, whereas q_e and q_t (mg g^{-1}) are the amount of phenol adsorbed at equilibrium and at any time, respectively. According to the plot of $ln(q_e-q_t)$ versus t, the k_1 and q_e were calculated by the slope and intercept.

$$ln(q_e - q_t) = lnq_e - k_1 t \quad \text{(S1)}$$

The pseudo-second order [59] model is described as (Equation S2): where k_2 (g mg^{-1} h^{-1}) is the second-order rate constant, and q_e and q_t (mg g^{-1}) have the same meaning described above. The values of k_2 and q_e were calculated from the slope and intercept of the linear plot of t/q_t versus t.

$$\frac{t}{q_t} = \frac{1}{k_2 q_e^2} + \frac{t}{q_e} \quad \text{(S2)}$$

Both models can be derived from the Langmuir kinetic equation depending on the conditions under which one or the other is obtained [54]. A (simplified) interpretation is that when the ratio of adsorbate to active sites is high, PSO is obtained, otherwise PFO.

Elovich Equation´s is commonly used to describe adsorption of gases, but it has also been used for describing the sorption of solid contaminants in aqueous solution. The basic assumptions [50] of the Elovich model were: a) the activation energy increased with adsorption time and b) the surface of the adsorbent was heterogeneous. Was used initially to describe chemisorption. It is applicable far from equilibrium and has been shown to be equivalent to the PSO model with surface coatings below 70%. The model is described at (Equation S3). Incorporates a as the speed initial adsorption (mg g^{-1} min^{-1}) and b (g mg^{-1}) as range-related desorption constant of the surface.

$$q_t = \frac{1}{b} ln(1 + abt) \quad \text{(S3)}$$

Natarajan and Khalaf [60] propose a first-order model that relates the initial concentration of the adsorbate (C_i) to its concentration at different times (C_t). The equation is expressed as (Equation S4):

$$log\frac{C_i}{C_t} = \frac{k}{2,303}t \quad (S4)$$

Where C_i (mg L^{-1}) is the initial concentration of adsorbate and C_t (mg L^{-1}) the concentration at time t. The value of k (h^{-1}) was calculated from the slope of the line obtained by the plot of log (C_i/C_t) versus t.

Bhattacharya and Venkobachar [61] presented a simple first order reversible kinetic model (Equation S5), based on solution concentration to study the mechanism of adsorption and characteristic constants of adsorption [59].

$$log\left[1 - \frac{C_i - C_t}{C_i - C_e}\right] = -\frac{k\,t}{2,303} \quad (S5)$$

Where C_i, C_t and C_e (mg L^{-1}) are the concentration initial, at the time t and at the equilibrium and k is the constant (h^{-1}).

Bangham's model equation is generally expressed as [62] (Equation S6):

$$loglog\left(\frac{C_i}{C_i - q_t\,m}\right) = log\left(\frac{k\,m}{2,303\,V}\right) + alog(t) \quad (S6)$$

Where, m the weight of adsorbent used per liter of solution (g L^{-1}), and a (<1) and k are constants.

If the experimental data are fitted to this equation thus the adsorption kinetics is limited by the pore diffusion [63].

The intraparticle diffusion kinetic model proposed by Weber and Morris [64] was widely applied to study the adsorption process to predict the rate-controlling step (Equation S7).

$$q_t = k\,t^{\frac{1}{2}} \quad (S7)$$

Where k (mg g^{-1} $h^{-1/2}$) is the intra-particle diffusion rate constant.

If the q_t versus $t^{1/2}$ was linear, intraparticle diffusion was involved and if the line passed through the origin, then the intraparticle diffusion was the only rate limiting process [63]. If this is not the case, other factors may affect the adsorption rate.

The film diffusion mass transfer rate equation presented by Boyd [65] is (Equation S8):

$$ln\left(1 - \frac{q_t}{q_e}\right) = -kt \quad (S8)$$

where k (h^{-1}) is liquid film diffusion constant.

A plot of ln(1−qt/qe) versus t should be a straight line with a slope k if the film diffusion is the rate limiting step [66].

References:

1. Lagergren, S.K. About the Theory of So-Called Adsorption of Soluble Substances. *Sven. Vetenskapsakad. Handingarl* **1898**, *24*, 1–39.
2. Ho, Y.S.; McKay, G. Pseudo-Second Order Model for Sorption Processes. *Process Biochemistry* **1999**, *34*, 451–465, doi:10.1016/S0032-9592(98)00112-5.
3. Kannan, N.; Vanangamudi, A. A Study on Removal of Cr(VI) by Adsorption Lignite Coal. *Indian Journal of Environmental Protection* **1991**, *114*, 241–245.
4. Bhattacharya, A.K.; Venkobachar, C. Removal of Cadmium (II) by Low Cost Adsorbents. Journal of Environmental Engineering 1984, 110, 110–122, doi:10.1061/(ASCE)0733-9372(1984)110:1(110).
5. Mall, I.D.; Srivastava, V.C.; Agarwal, N.K. Removal of Orange-G and Methyl Violet Dyes by Adsorption onto Bagasse Fly Ash—Kinetic Study and Equilibrium Isotherm Analyses. Dyes and Pigments 2006, 69, 210–223, doi:10.1016/J.DYEPIG.2005.03.013.
6. Mohamed Nasser, S.; Abbas, M.; Trari, M. Understanding the Rate-Limiting Step Adsorption Kinetics onto Biomaterials for Mechanism Adsorption Control. Progress in Reaction Kinetics and Mechanism 2024, 49, doi:10.1177/14686783241226858.
7. WeberJr., W.J.; Morris, J.C. Kinetics of Adsorption on Carbon from Solution. Journal of the Sanitary Engineering Division 1963, 89, 31–59, doi:10.1061/JSEDAI.0000430.
8. Boyd, G.E.; Adamson, A.W.; Myers, L.S. The Exchange Adsorption of Ions from Aqueous Solutions by Organic Zeolites. II. Kinetics. J Am Chem Soc 1947, 69, 2836–2848, doi:10.1021/JA01203A066.
9. Qiu, H.; Lv, L.; Pan, B.C.; Zhang, Q.J.; Zhang, W.M.; Zhang, Q.X. Critical Review in Adsorption Kinetic Models. Journal of Zhejiang University: Science A 2009, 10, 716–724, doi:10.1631/JZUS.A0820524/METRICS.
